# A Comparison of the Distortion in the Same Field MRI and MR-Linac System With a 3D Printed Phantom

**DOI:** 10.3389/fonc.2021.579451

**Published:** 2021-06-03

**Authors:** Xuechun Liu, Zhenjiang Li, Yi Rong, Minsong Cao, Hongyu Li, Chuntao Jia, Liting Shi, Weizhao Lu, Guanzhong Gong, Yong Yin, Jianfeng Qiu

**Affiliations:** ^1^ Medical Engineering and Technology Research Center, Department of Radiology, Shandong First Medical University & Shandong Academy of Medical Sciences, Tai’an, China; ^2^ Department of Radiation Physics, Shandong Cancer Hospital and Institute, Shandong First Medical University and Shandong Academy of Medical Sciences, Jinan, China; ^3^ Department of Radiation Oncology, University of California Davis Comprehensive Cancer Center, Sacramento, CA, United States; ^4^ Department of Radiation Oncology, David Geffen School of Medicine, University of California, Los Angeles, CA, United States; ^5^ Academy of Marine Science and Engineering, Shandong University of Science and Technology, Qingdao, China

**Keywords:** geometric distortion, 3D printed phantom, magnetic resonance imaging-linac, magnetic resonance imaging, radiotherapy

## Abstract

**Purpose:**

A 3D printed geometric phantom was developed that can be scanned with computed tomography (CT) and magnetic resonance imaging (MRI) to measure the geometric distortion and determine the relevant dose changes.

**Materials and Methods:**

A self-designed 3D printed photosensitive resin phantom was used, which adopts grid-like structures and has 822 1 cm^2^ squares. The scanning plan was delivered by three MRI scanners: the Elekta Unity MR-Linac 1.5T, GE Signa HDe 1.5T, and GE Discovery-sim 750 3.0T. The geometric distortion comparison was concentrated on two 1.5T MRI systems, whereas the 3.0T MRI was used as a supplemental experiment. The most central transverse images in each dataset were selected to demonstrate the plane distortion. Some mark points were selected to analyze the distortion in the 3D direction based on the plane geometric distortion. A treatment plan was created with the off-line Monaco system.

**Results:**

The distortion increases gradually from the center to the outside. The distortion range is 0.79 ± 0.40 mm for the Unity, 1.31 ± 0.56 mm for the GE Signa HDe, and 2.82 ± 1.48 mm for the GE Discovery-sim 750. Additionally, the geometric distortion slightly affects the actual planning dose of the radiotherapy.

**Conclusion:**

Geometric distortion increases gradually from the center to the outside. The distortion values of the Unity were smaller than those of the GE Signa HDe, and the Unity has the smallest geometric distortion. Finally, the Unity’s dose variation best matched with the standard treatment plan.

## Introduction

Radiation therapy aims to maximize the delivered dose to tumor, while sparing normal tissue. Image-guided radiation therapy (IGRT) takes into account that changes in tumor size and shape during therapy may occur and allows for adapted treatment plans based on same-day tumor localization and volume measures. Most IGRT devices are based on cone beam computed tomography (CBCT) (1). However, compared with CT, magnetic resonance imaging (MRI) has the advantage of having a superior soft-tissue contrast. The integration of MRI systems and a linear accelerator (linac) has promoted IGRT development (2) and attracted more attention (3–7).

Although MRI has unique advantages, it also has evident shortcomings, such as geometric distortions, signal dropout, and artifacts. Once there is sudden interference, the image quality is affected (8). Geometric distortion is an important factor in MRI-guided radiation therapy (9). The geometric distortion affects the original images, which affects the delineation of the gross tumor volume (GTV) and clinical target volume (CTV), thus ultimately affecting the treatment plans and dose delivery.

The geometric distortion can be classified as patient-dependent and system-dependent (10, 11). The system-dependent distortions are related to the MRI hardware, such as the main magnetic field and the gradient field uniformity and linearity. The patient-dependent distortions are related to magnetic properties such as magnetic susceptibility and chemical shift effects. The patient-dependent distortion is smaller in magnitude than the system-dependent distortion but more difficult to correct (12). The geometric distortions must be measured and corrected before delineating the GTV. The geometric distortion is measured using regular-shaped phantoms (13, 14) that contain square grids and cylindrical rods.

A variety of commercial phantoms have been developed. When considering 2D measurements, the common phantoms include the American College of Radiology (ACR) phantom and the spatial integrity phantom (Fluke Biomedical, Everett, WA). For the 3D-measurements, the common phantoms include the MAGPHAN^®^ phantom series (The Phantom Laboratory, Greenwich, NY, USA). However, commercial phantoms are complicated to manufacture and expensive as well. Consequently, we designed a 3D grid-like structure phantom to measure the large field of view (FOV) geometric distortion since 3D printing technology is widely applied (15–17), and we can calculate the relevant dose changes.

## Materials and Methods

### 3D-Printed MRI Testing Phantom

**Figure 1:**
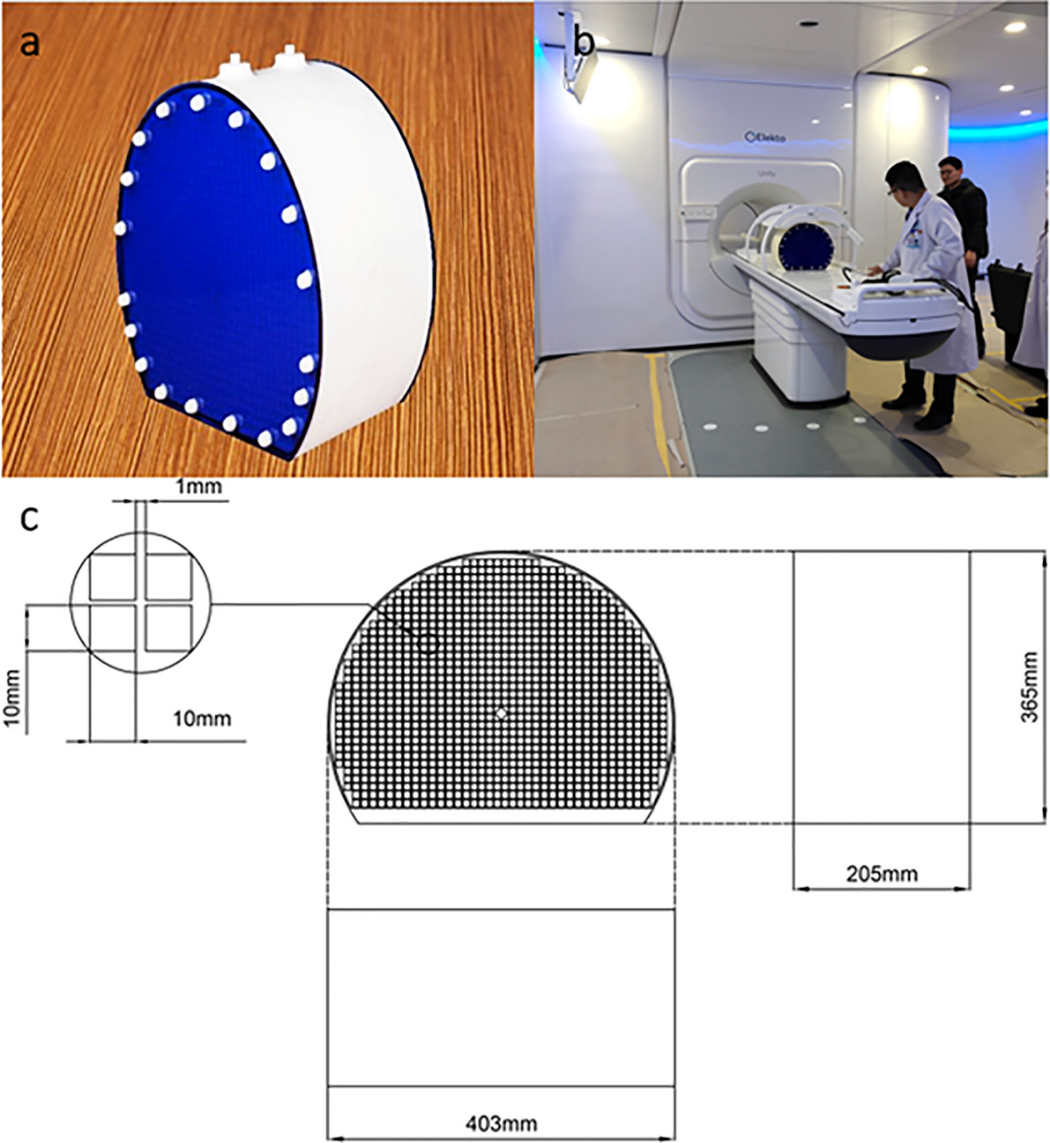
Realistic photo of the 3D printed phantom **(A)**. The placement of the phantom in Unity before image scanning **(B)**. The detailed size and structural design of the 3D printed phantom **(C)**.

Using the full 3D printing method, we developed a photosensitive resin phantom with a Union Tech Lite 600HD printer. Before adding water, the phantom weighed 10.7 kg, and after adding water, it weighed 24.9 kg. The front side of the phantom is a circle with a diameter of 40 cm, and it has been partly cut off for stability. The side of the phantom is similar to the cylindrical side. The phantom adopts grid-like structures, and its interior contains 822 1 cm^2^ grid structures. The purpose of designing 822 small squares is to provide more standard geometric structures. A realistic photograph and detailed structural design are shown in **Figure 1**.

In the transverse direction, the phantom center was designed as a diamond to precisely position the image. The overall thickness of the phantom was 22 cm. On the outside, there were 38 external hexagonal bolts for fixation and two 7-mm diameter water injection ports on the top for the injection of the copper sulfate solution. Another positioning design is the crosshair, which was on the outside surface to register the laser before scanning.

### Scanning Systems

MRI data were acquired using the Elekta Unity MR-linac (Sweden, 1.5T, with a built-in body coil) and a 1.5T scanner (GE Signa HDe, with a built-in body coil). A 3.0T scanner (GE Discovery-sim 750, with the built-in body coil) was also used to measure the geometric distortion. In this study,

The comparison was mainly between the Unity and GE Signa HDe, and the GE Discovery-sim 750 was regarded as a supplementary experiment.

Considering the printing precision of 3D printing technology and the shape change of the water phantom, the CT images were regarded as the gold standard. In addition, the body coil was applied in the scanning process because the large FOV phantom ran out of the other coil sizes. The scanning line was aligned with the phantom side mark. The central diamond was taken as the center point to obtain the transverse, coronal, and sagittal images. When considering the image quality, the T2 sequences were selected for scanning.

The Unity system combines a 1.5T split bore MR scanner with a radiation therapy gantry, which was produced by Philips and Elekta. Concretely, the default scanning parameters that were selected have a FOV of 537.6 mm × 537.6 mm, TR of 2100 ms, TE of 220 ms, resolution of 1008×1008 pixels, and pixel bandwidth of 1033 Hz/pixel. In addition, to ensure that the sequence parameters were consistent with the other two systems, we scanned the unified sequence with the same bandwidth and resolution. The scanning series of the Unity consisted of a 3D series; hence, the layer thickness was not involved.

The 1.5T GE Signa HDe is a diagnostic MRI system that is used in clinical diagnostic medical imaging. The scanning parameters of the GE Signa HDe have a FOV of 480 mm × 480 mm, TR of 3000 ms, TE of 81.6 ms, resolution of 512×512 pixels, pixel bandwidth of 195.31 Hz/pixel, and layer thickness of 5 mm.

The GE Discovery-sim 750 system was applied for simulation in radiotherapy. It is an independent MRI system and has a flatbed and a large body coil for radiotherapy planning. The scanning parameters of the GE 750 have a FOV of 500 mm × 500 mm, TR of 13051 ms, TE of 139 ms, resolution of 512×512 pixels, pixel bandwidth of 195.31 Hz/pixel, and layer thickness of 3 mm. However, notably, the acquisition parameters were not changed to optimize the image quality or to minimize the distortion across the three different scanners. The same scanning position ranges were selected to make the sequences as consistent as possible. However, different vendors tend to have different specific parameters, and the subsequent comparison and the analysis of the data were performed by focusing on the data collected in this study.

### Image Analysis

**Figure 2:**
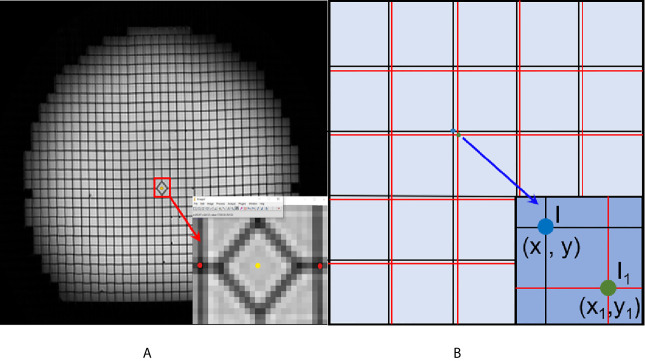
Schematic diagram of the geometric distortion obtaining process. **(A)** The center point (yellow point) and distortion points (red points) in the enlarged image. **(B)** The registered CT and MR image. The black grids represent the reference CT image, and the red line represents the MR image.

In this experiment, the geometric distortions were characterized for the MR images acquired using the vendor-supplied distortion correction algorithms that were applied. Residual geometric distortion was used for the distortion analysis in this study. ImageJ (https://imagej.en.softonic.com/) was used as an image-processing software. Transverse images were selected to demonstrate the plane distortion. To reduce the experimental error, the most central transverse image of every dataset was selected to record the point coordinates. A schematic is shown in **Figure 2**. First, we enlarged the transverse image 32 times. We then acquired the coordinate values of the distortion points, as indicated by the red points. The yellow point represents the center point in the plane. Further, we registered the center point of the CT image and the MR image due to the smallest distortion at the central point. Finally, the horizontal and vertical coordinates of the registered MR image were subtracted from the CT image, and the distance between the two points was defined as the plane distortion value of the point. The distortion value is obtained using the distance formula.

$$The\;plane\;distortion=\sqrt{{\left(x1-x\right)}^{2}+{\left(y1-y\right)}^{2}}$$

(*x, y*) represents the x and y coordinates of the CT image. (*x_1_*, y_1_) represents the x and y coordinates of the CT image.

**Figure 3:**
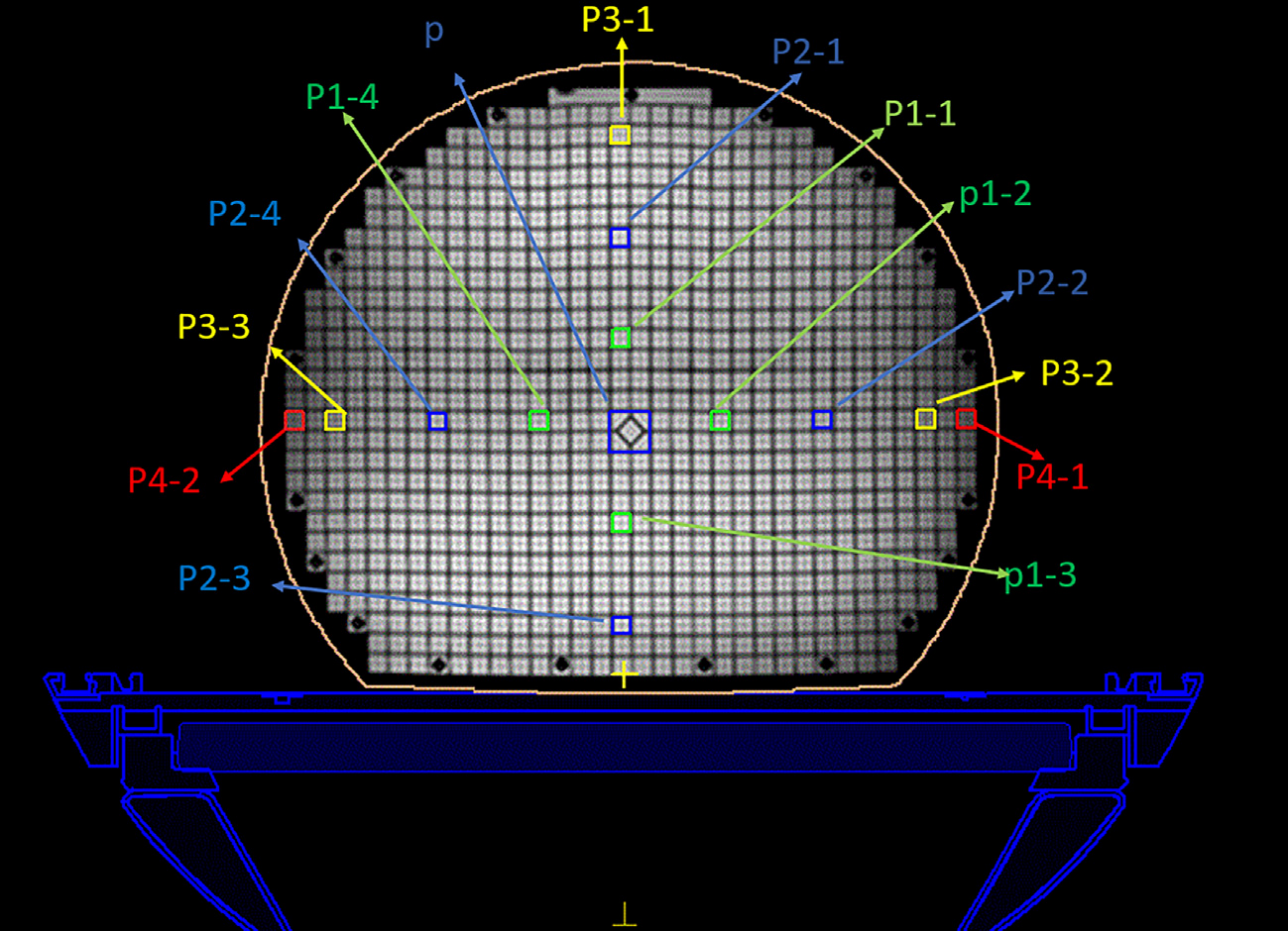
Regions of interest of the MRI image. The center diamond square is regarded as the central point. The green, blue, and yellow circle represent the 5 cm, 10 cm, and 15 cm ranges, respectively.

A 3D geometric distortion analysis was also performed using the ImageJ software. The central point of the diamond square was defined as that of the phantom. In the plane, 5, 10, and 15 cm ranges are defined in the transverse image. The range is shown in **Figure 3**. The range definition was based on the distance between the center point and the distortion point. Owing to the large number of distortion points, twelve mark points in the plane were selected to analyze the distortion in the 3D direction based on the plane geometric distortion. The positive direction of the y-axis is defined as 0°, and the points are sequentially defined as 90°, 180°, and 270° in a clockwise direction. In the 15 cm range, the 180° point is not excited in the phantom image because of the limitation of the phantom size. The coordinate information of each marked point in each layer was easily obtained. The scanning layers of the three machines are different, and there was a minimum of 30 layers. Because the scanning layers of the three groups of images are multiples of 30, we chose the layer closest to the foot direction as the bottom layer. For the other layers, the layer closest to the head direction was chosen as the selected layer.

The change in the mark points in the head and foot direction was calculated by subtracting the next foot direction layer from this layer. The change in the distance between the points between the two layers is defined as the change in the direction of the head and foot. Finally, the 3D geometric distortions were defined as the coordinate change in the plane, head, and foot directions. The specific distortion value was obtained using a 3D distance formula.

$$3D\;distortion=\sqrt{{\left(x1-x\right)}^{2}+{\left(y1-y\right)}^{2}+{\left(z1-z\right)}^{2}}$$

(*x, y, z*) represents the x-, y-, and z-coordinates in the plane close to the foot. (*x_1_*, *y_1_)* represents the x- and y-coordinates in the plane close to the head.

### Treatment Planning

The treatment plans were developed in the offline Monaco system (Elekta Radiation Oncology Systems) version 5.40.01. To quantify the dose difference by the geometric distortions, the MR phantom images from each scanner were registered with the CT reference images. A rigid registration method was applied to compensate for the geometric changes between the different modalities, and a mutual information registration algorithm was used to account for the nonlinear MRI deformations. After image registration, four different datasets were generated: the CT reference images and three MR datasets from the different devices.

By focusing on this self-designed phantom, a prescription of 50 Gy in accordance with the standard clinical protocol treatment plan was specified for the dose calculation. According to the different distances from the center point, 14 regions of interest were selected in every transverse image, as shown in **Figure 3**. The central area (p) was defined as a 2 × 2 cm^2^, another area was defined as a 1 × 1 cm^2^, and the distances from the center point were 5 cm (p1), 10 cm (p2), 15 cm (p3), and 17 cm (p4). The p4 points are the edge points of the phantom. The dose distributions were then evaluated using the standard of a clinically acceptable DVH and the assessment criteria.

The static IMRT plans were optimized by adjusting the appropriate IMRT optimization parameters and visually accepted by an experienced senior planner. The original IMRT plan was generated in the CT dataset, and it propagated to the other three MRI datasets. In this process, the optimization parameters remained consistent to avoid variations in the planning techniques. Senior planner visual acceptance was based on the department’s clinical practice, and it ensured an acceptable assessment of the 100% and 95% isodose curves compared to the DVH criteria. By focusing on the influence of the magnetic field, the treatment plan adopted the same conditions while only changing the magnetic field; thus, every region of interest received eight different radiation therapy doses.

### Commercial Phantom

During this experiment, a commercial phantom was used with the Unity system to evaluate the geometric distortion. The geometric distortion of the MR-Linac was characterized on a large (500 × 375 × 330 mm^3^) geometric fidelity phantom provided by Philips. The phantom contains 1,932 markers spaced 25 mm × 25 mm × 55 mm apart, which performed well on magnetic resonance imaging (18). Unity has specialized scanning modes and distortion analysis software for commercial phantoms. We scanned this commercial phantom using Unity and compared results with our self-designed phantom results to verify the accuracy of our 3D printed phantom.

## Results

### Plane Distortion

The center layer image was used to describe the detailed distortion of each point. After analyzing the three datasets, it intuitively shows that every dataset has a different distortion value range. In simple terms, each dataset contains 822 coordinate points and 822 distortion values. We calculated the actual distortion value by using an in-house developed image analysis program that was implemented in MATLAB. After we changed the bandwidth from 1033 Hz/pixel to 195.31 Hz/pixel, the mean distortions increased slightly from 0.79 mm to 0.85 mm and the maximum distortion increased from 2.37 to 2.44 mm.

**Figure 4:**
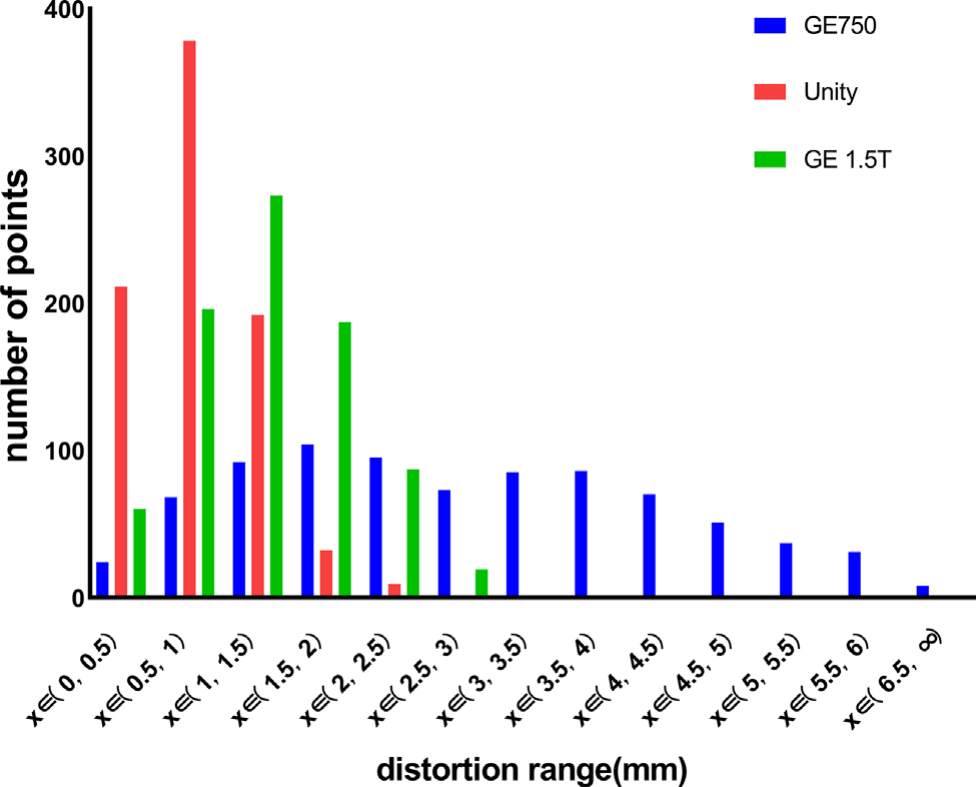
Distribution range of the distortion points for GE 750, Unity, and the GE Signa HDe.

**Figure 4** displays the GE Discovery-sim 750, which generates the largest distortion range (0–6.5 mm), while the distortion point range of the Unity and GE Signa HDe are between 0–2.5 mm and 0–3 mm, respectively. Otherwise, the most numerous of the distortion points in the Unity and GE Signa HDe are 0.5–1 mm and 1–1.5 mm, respectively. In general, the distortion ranges of the Unity and GE Signa HDe are concentrated between 0 and 2.5 mm, while strictly speaking, the most concentrated ranges of Unity and GE Signa HDE are 0–2 mm and 0–2.5 mm, respectively. This indicates that the distortion range of the GE Signa HDe is a bit larger than Unity in this experiment. Furthermore, distortions on the 3.0T GE Discovery-sim 750 are expected to be intrinsically more severe than on a 1.5T scanner.

**Figure 5:**
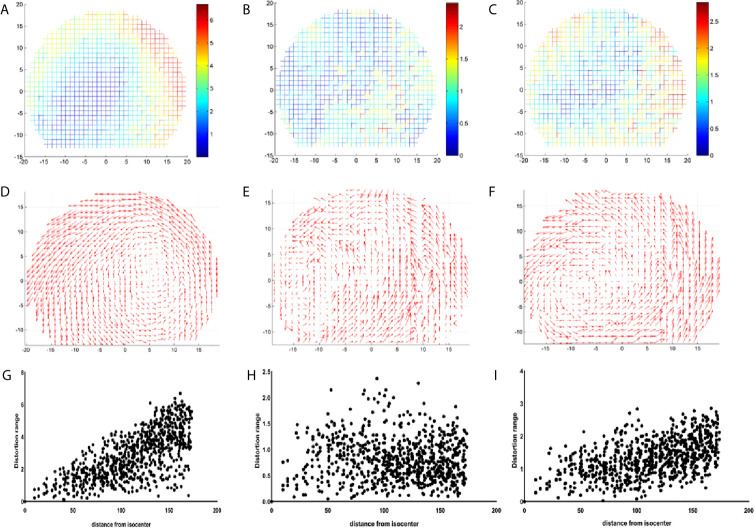
Coordinate diagram of the distribution of the distortion points for GE 750 **(A)**, Unity **(B)**, and GE Signa HDe **(C)**. The vector of the distortion points for GE 750 **(D)**, Unity **(E)**, and GE Signa HDe **(F)**. The scatter diagram of the distribution of the distortion points for GE 750 **(G)**, Unity **(H)**, and GE Signa HDe **(I)**.

**Figures 5A–C** visually shows the actual size of each distortion point. The x-axis and y-axis represent the actual length of the phantom and the origin of the coordinate is the diamond mesh of the phantom. The color of the distortion points represents the size of the distortion value. From blue to red, the distortion value increases from a small value to a large value. The distortion distribution of the GE Discovery-sim 750 is typical. The closer it is to the central point, the smaller the distortion value is. It can be observed that the range of the distortion points range for the GE Discovery-sim 750 gradually changed, and the changes are relatively regular. This means that the distortion of the GE Discovery-sim 750 is relatively stable, and the large distortion is far away from the center and on the outside of the phantom.

For Unity, the most central distortion point is blue, and the overall color is closest to blue, whether it is inside or on the outside. It can be observed that the variation of the distortion is relatively uniform, and the distortion range is mainly concentrated at 0 to 2.5 mm. Although there is some yellow in the border position, the overall distortion is still characterized by a small intermediate distortion and a large peripheral distortion.

For the GE Signa HDe, from the center to the boundary, the distortion points are blue, light blue, yellow, and green, and they are arranged in concentric circles. This shows the uniform variation of the distortion points, and the number of distortion points in each distortion range is quite large. It is worth noting that in this figure, the maximum distortion value range is approximately 2.8 mm; therefore, the GE Signa HDe’s distortion value is close to the Unity devices.

**Figures 5D–F** show the vector difference of each point. The coordinate systems are the same as the coordinate system in **Figures 5A–C**. The size of the arrow indicates the size and direction of the distortion. The overall variation trend of the GE Discovery-sim 750 is intuitive. Visually, the distortion appears to increasing in magnitude from the center to the periphery. The vectors of the Unity and GE Signa HDe also shows the increase in general, but it is not as remarkable as the GE Discovery-sim 750.

**Figures 5G–I** present a series of scatter plots that shows the distributions of the measured geometric distortion for each of the phantom’s reference points. It demonstrates an increase in the geometric distortion when the distance from the isocenter increases gradually in the GE Discovery-sim 750 and GE Signa HDe. In Unity, the geometric distortion does not have an obvious pattern, which may be attributed to the small and uniform geometric distortion.

The distributions of the distortion values for the three devices are shown in **Table 1**. **Table 1** presents the average distortion values and standard deviations of the three devices for different radius ranges. The average distortion value can be easily obtained by sorting and analyzing the above data. The distortion range of the GE Discovery-sim 750 is 2.82 ± 1.48 mm, the distortion range of the Unity is 0.79 ± 0.40 mm, and the maximum longitudinal distortion value of the GE Signa HDe is 1.31 ± 0.56 mm. In general, the average distortion values of GE Signa HDe and Unity are close, whereas the average distortion values of GE Discovery-sim 750 are larger in magnitude.

**Table 1:** Maximum distortion values and the standard deviation of the three devices in the different radius ranges, respectively.

Devices	GE750		Unity		GE 1.5T	
	Mean	SD	Mean	SD	Mean	SD
5 cm	0.84	0.44	0.69	0.41	0.78	0.40
10 cm	1.70	0.94	0.84	0.46	1.05	0.52
15 cm	1.97	1.00	0.80	0.43	1.09	0.50
20 cm	2.80*	1.48	0.79	0.40	1.31	0.56

*The symbol in the table represents a distortion larger than 2 mm.

### Three-Dimensional Distortion

**Figure 6:**
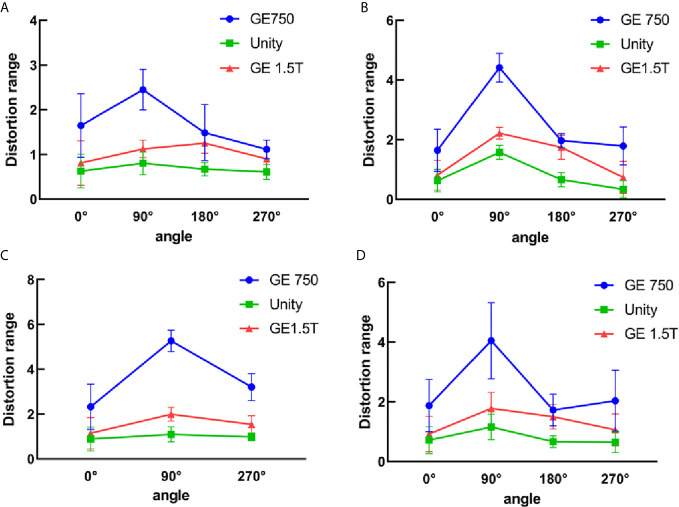
3D distortion between GE750, Unity, and GE 1.5T for the different distance ranges. **(A)** 5 cm from the central point, **(B)** 10 cm from the central point, **(C)** 15 cm from the central point, and **(D)** all the distortion points within 15 cm.

The three-dimensional geometric distortions of the mark points are shown in **Figure 6**. Overall, the Unity and GE Signa HDe have similar distortions at multiple mark points. The variation rule of the distortion of these two devices is consistent in every statistical graph. However, the distortion of the GE Signa HDe is larger than Unity at the same mark point, regardless of the range. The distortion curve of GE Discovery-sim 750 is higher than the two machines. This means that the distortion of GE Discovery-sim 750 is significantly greater than the two machines. As the distance from the center increases, the distortion increases. In addition, at the same distance from the center point, the distortion in the 90° angle direction is larger than that at the other angles.

The mean 3D distortion values and standard deviations of the three devices for different radius ranges and angles are listed in **Table 2**. The self-designed phantom was simulated as a miniaturized and same-ratio phantom. For the 3D distortion calculation, the distortion of the head and foot direction was added in comparison to the plane distortion. According to the table analysis, the 3D distortion rule is consistent with the plane distortion law. It is intuitive to show that the 3D distortion increases with the distance from the origin. The overall distortions of GE Discovery-sim 750 are still greater than those of the two 1.5T systems. Under the same range and angle, the distortion of Unity is smaller than the GE Signa HDe.

**Table 2:** Mean 3D distortion values and the standard deviation of the three devices for the different radius ranges and angles, respectively.

Devices		GE750		Unity		GE 1.5T	
Distance	Angle	Mean	SD	Mean	SD	Mean	SD
5 cm	0°	1.65	0.71	0.63	0.38	0.81	0.50
	90°	2.45*	0.46	0.80	0.26	1.13	0.20
	180°	1.49	0.63	0.67	0.15	1.26	0.23
	270°	1.11	0.21	0.61	0.17	0.90	0.24
	average	1.68	0.50	0.68	0.24	1.02	0.29
10 cm	0°	1.65	0.71	0.63	0.38	0.81	0.50
	90°	4.42*	0.49	1.58	0.24	2.23*	0.20
	180°	1.97	0.24	0.66	0.24	1.75	0.41
	270°	1.80	0.64	0.34	0.29	0.74	0.54
	average	2.46*	0.52	0.80	0.29	1.38	0.41
15 cm	0°	2.33*	1.01	0.89	0.54	1.15	0.71
	90°	5.27*	0.48	1.10	0.34	2.00*	0.31
	270°	3.21*	0.60	0.99	0.18	1.54	0.39
	average	3.60*	0.70	0.99	0.35	1.56	0.47

*The symbol in the table represents a distortion larger than 2 mm.

### The Dose Differences

**Figure 7:**
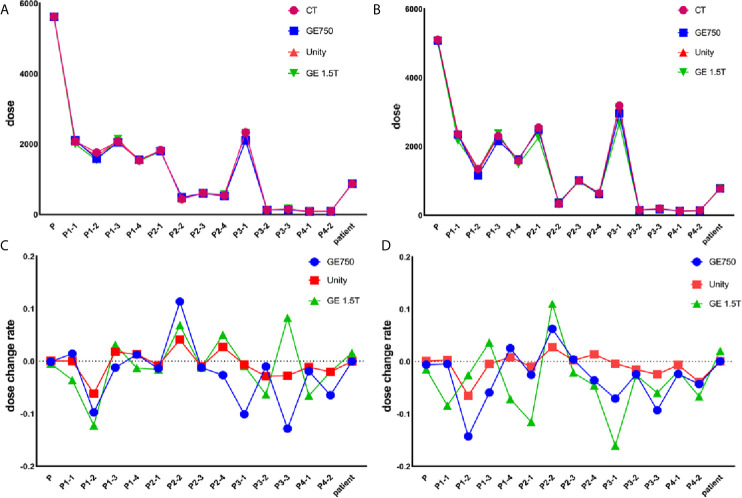
Actual dose without the magnetic field **(A)** and in the magnetic field **(B)**. The dose change rate without the magnetic field **(C)** and in the magnetic field **(D)**.

**Table 3** presents the mean dose differences of the CT, GE Discovery-sim 750, Unity, and GE Signa HDe in the magnetic field and without the magnetic field. It contains 16 regions of interest and an integral dose of the entire phantom. Both **Table 3** and **Figure 7** demonstrate a broadly consistent dose distribution. The dose difference is negligible when the entire dose of the treatment plan is high. There was no significant difference between the actual dose of the region of interest and the standard. In the case of a magnetic field and without a magnetic field, the actual dose in the region of interest is slightly different, but the difference is small.

**Table 3:** Dose change rate of GE 750, Unity, and GE 1.5T in comparison to the CT image dose in the magnetic field and without the magnetic field.

Devices	Change in mag condition (%)			Change in nomag condition (%)		
	GE 750	Unity	GE 1.5T	GE 750	Unity	GE 1.5T
p	0.58	0.10	1.46	0.09	0.08	0.58
P1-1	0.46	0.30	8.38	1.48	0.05	0.46
P1-2	14.27*	6.50	2.55	9.72	6.13	14.27*
P1-3	5.91	0.44	3.68	1.20	1.82	5.91
P1-4	2.57	0.84	7.13	1.27	1.33	2.57
P2-1	2.53	0.98	11.51*	1.37	0.75	2.53
P2-2	6.28	2.77	11.04*	11.39*	4.16	6.28
P2-3	0.43	0.22	2.08	1.20	0.99	0.43
P2-4	3.58	1.38	4.55	2.62	2.77	3.58
P3-1	7.06	0.39	16.03*	10.07*	0.65	7.06
P3-2	2.42	1.51	2.56	1.00	2.84	2.42
P3-3	9.28	2.44	5.96	12.82*	2.74	9.28
P4-1	2.38	0.63	1.83	1.88	1.11	2.38
P4-2	4.30	3.88	6.66	6.45	2.01	4.30
Patient	0.05	0.08	2.08	0.02	0.01	0.05

*The symbols in the table represent the dose change rate larger than 10%.

For the treatment plan without the magnetic field, the difference values of the GE Discovery-sim 750 from the CT gold standard are higher than the others, with an emphasis on P3-1. In the magnetic field treatment plan, the difference values of the GE Signa HDe from the CT gold standard are higher than those for Unity and Discovery-sim 750, with an emphasis on P2-1 and P3-1. The dose change rate for GE Signa HDe is the highest, followed by GE Discovery-sim 750, and the lowest dose change rate is Unity. The dose in the absence of a magnetic field is closer to the delivery dose due to the electron return effect. In addition, the dose rates of GE Discovery-sim 750 and GE Signa HDe were nearly the same, whereas the dose rate for Unity is lower than both of them.

### Distortion in the Commercial Phantom

The distortion result was based on the distortion analysis algorithm. We chose the maximum and 98% percentage points of the Total/RL/AP/FH distortion to represent the commercial phantom distortion. There was a coincidence in the distance from the center points of the two phantoms. Based on **Tables 1** and **4**, it can be observed that the distortion ranges of the two individual phantoms are relatively close. When the radius range is 10–20 cm, the average distortion of the two phantoms is within the range of 1.29 mm. In the range of 15 cm, the mean distortion is smaller than 1 mm.

**Table 4:** Distortion of the commercial phantom.

98% perc	Total (mm)		RL (mm)		AP (mm)		FH (mm)	
	max	98% perc	max	98% perc	max	98% perc	max
10 cm	0.51	0.53	0.24	0.26	0.41	0.43	0.39	0.43
15 cm	0.78	0.85	0.31	0.42	0.71	0.77	0.47	0.51
20cm	1.29	1.58	0.61	0.86	1.06	1.32	0.72	0.83
25cm	2.55	8.28	0.99	3.49	2.28	7.51	0.89	1.35

Total/RL/AP/FH (98%) is the maximum measured distortion [mm] after discarding 2% of the markers with the largest total/RL/AP/FH distortion.

## Discussion

Geometric distortion phantoms are widely used in clinical applications. Vendors provide commercial phantoms and algorithms to decrease the geometric distortions (19). Wang et al. applied an ACR phantom to test the geometric distortion, slice thickness accuracy, and the image uniformity (20). Ginn et al. used two commercially available phantoms to comprehensively assess the spatial distortion of a 0.35 T MRI-guided radiotherapy system (21). The ACR phantom can measure the geometric distortion, gradient/radiofrequency (RF) subsystem, the percent image uniformity (PIU), and other ordinary MRI scanner imaging parameters (22).

Self-designed geometric distortion phantoms have a considerable history. More recently, Wang et al. (23) designed a cube-shaped phantom that consists of a grid of orthogonal acrylic planes, and the space of the planes was filled with an orthogonal grid of acrylic planes filled with aqueous solutions. In addition, Rai et al. designed a 3D printed geometric distortion phantom without water filling (24). The 3D printed MRI visible materials have great potential for use as MRI phantoms. The phantom addressed the disadvantages for the traditional MRI phantom construction, which include susceptibility artifacts and an increased weight due to filling. However, MRI-visible materials are relatively new materials, and our material is more common and cheaper. Grid structures are often used to measure the traditional geometric deformation modes, and our self-designed 3D printed phantom also adopted the grid structures. In this study, the material that was used for the phantom was acrylic, the filling liquid was water, and the interior design consisted of a large number of grid structures.

Geometric distortion phantoms have a strong practicability due to their regular shape, but they are limited by their actual size. If the size of a measured area is larger than the phantom, the geometric distortion message will be less than desired. To solve this problem, the phantom we designed has a larger volume and more detection points; thus, it achieves a higher detection accuracy. In addition, the price of the traditional commercial phantom is quite high for a normal hospital, and although its advantages of high repeatability are obvious, the frequency of its actual application in practice depends on hospital requirements. By combining the cost and application frequency, the cost of a 3D printed phantom is lower than a commercial phantom, and the application frequencies of the two phantoms are similar. One of the obvious advantages of a 3D printed phantom is that the size of the phantom is controllable and it can be adapted depending on the actual requirement.

The distortion distribution shows that the geometric distortion increases gradually from the center to the outside (18). The three-dimensional geometric distortions of the mark points were consistent with the plane geometric distortions. In the case of a relatively regular distortion, the distortion for the GE Discovery-sim 750 is typical. The system-dependent distortions are related to the main magnetic field (11), the gradient field uniformity, and the linearity. After applying the correction from the algorithm that was provided by the supplier, the residual distortion of the 3.0T magnetic resonance is larger than the other two 1.5T magnetic resonances. In addition, our organization also scanned the CIRS 008z simulated chest phantom by using different magnetic resonance scanners. For the simulated phantom, we did not observe any obvious geometric distortions.

A central transverse image was selected to calculate the distortion. According to the data analysis of the transverse images, there were 98.78% and 87.10% points for Unity and GE Signa HDe with distortion values that were less than 2 mm. By referencing the research of Dorsch et al. (19), the mean distortion over the whole phantom was 0.60 ± 0.28 mm and 99.80% of the evaluated control points had distortions below 1.5 mm. This may prove that the precision requirements for the geometric distortion of MRI-Linac are stricter than for the ordinary MR, although the scanning system and scanning sequence are similar to diagnostic MR scanners that are used in clinical applications (25). On the other hand, the 3D geometric distortion value of every mark point of Unity was smaller than GE Signa HDe. This also indicates that the geometric distortion specifications of MR-Linac are stricter than those of the ordinary MR scanners.

The commercial phantom also measures the distortion within a 25 cm radius, whereas the 3D printing phantom does not cover the 25 cm range. Therefore, we did not compare the magnitude of the geometric distortion within a radius of 25 cm. The distortion values in the 10- to 20-cm range were smaller than 2 mm, which is similar to our results. There are some errors between the two phantoms because the commercial phantom only selects 98% of the distortion points, while the 3D printing phantom geometric distortion calculation takes all the distortion points into account. In addition, the heights of the central points of the two modes are not the same, which may be one of the reasons for the different distortion ranges. Unity’s built-in geometric distortion analysis report shows that the distortion range of Unity meets the clinical standards and it indirectly proves the accuracy of our self-designed phantom.

In this study, we investigated the dose effects that are caused by the geometric distortion in a Monaco treatment planning system. In addition, because the lack of electron density information in MRI is another major impediment to MRI‐based treatment planning, we also considered the dose effects that are caused by the CT number assignment. The electron density was set to zero to avoid the dose effects of the MRI that do not possess electron density. All the CT and MRI plans were compared to determine the impact of the distortions on the dose distributions. The MR-Linac and MR-sim MR scanners performed similarly in terms of the dose impact with respect to the system distortion effects that were measured by the phantoms. In addition, Unity’s dose change rate is closest to the standard dose change rate. Therefore, we believe that Unity is more suitable for planning in the planning system, despite the known increase in the gradient nonlinearity distortions with the increased distance from the isocenter. The geometric distortions of the phantom also did not adversely impact the dosimetry. Other researchers also determined that system distortions have no significant impact on the dosimetry (26, 27).

GE Discovery-sim 750 had the largest geometric distortion, whereas its actual planned dose and dose rate were similar to the standard CT dataset. Han et al. (28) simulated a 2.77 mm average geometric distortion of the MRI with a phantom, and the average dose uncertainties for the target volumes and critical structures were small. The average distortion of the GE Discovery-sim 750 was 2.82 mm, which is close to Han’s result; thus, small distortions have minimal impact on the dosimetry for the MRI systems. In addition, several researchers have studied the effect of MRI spatial inaccuracies on treatment planning for prostate cancer. These can lead to dose differences of less than 2.0% between the MRI- and CT-based treatment plans (29–31).

The main aim of this study was to design and build a 3D printed geometric phantom that can be scanned by CT and MRI, followed by measuring the geometric distortion, and calculating the relevant dose changes. Although there have been many outstanding studies on geometric distortion phantoms and related issues, this study is unique in that it combined a geometric distortion analysis and dosimetric changes analysis. As a result, this study proved that Unity is the most suitable for IGRT among the three MR scanners. In the future, we will consider adding grid patterns in anatomical phantoms to achieve a stricter comparison of the clinical systems and protocols.

## Conclusion

From the center to the outside, the geometric distortion gradually increased. The final statistical analysis showed that the distortion values of Unity are smaller than those of the GE Signa HDe. This may prove that the precision requirements for the geometric distortion of MRI-Linac are stricter than an ordinary MR scanner. By comparing the geometric distortion of the commercial phantom, our results indicate that our phantom is sensitive enough to detect a variability in the performance between the different MRI systems in the geometric distortion test. In addition, the geometric distortion that meets the clinical application criteria has less impact on the treatment planning. For these three devices, Unity has the smallest geometric distortion and the dose variation that best matches the standard treatment plan.

## Data Availability Statement

The original contributions presented in the study are included in the article, further inquiries can be directed to the corresponding authors.

## Author Contributions

JQ, CJ, WL, and LS conceptualized and designed the phantom. ZL and XL coordinated and supervised the experimental process. YY and GG helped with the data collection. ZL and XL analyzed and interpreted the clinical data and performed the final data analyses. XL drafted the manuscript. MC and YR critically reviewed and revised the manuscript. All authors contributed to the article and approved the submitted version.

## Funding

We wish to thank the funding support from the National Key Research and Development Program (2016YFC0103400) and the Natural Science Foundation of Shandong Province (grant no. ZR2018BH028, ZR2016HP32). In addition, JQ was supported by the Taishan Scholars Program of Shandong Province (no. TS201712065).

## Conflict of Interest

The authors declare that the research was conducted in the absence of any commercial or financial relationships that could be construed as a potential conflict of interest.
